# Complex Mosaicism of Two Distinct Mutations in a Female Patient With *KCNA2*-Related Encephalopathy: A Case Report

**DOI:** 10.3389/fgene.2020.00911

**Published:** 2020-08-12

**Authors:** Pan Gong, Xianru Jiao, Yuehua Zhang, Zhixian Yang

**Affiliations:** Department of Pediatrics, Peking University First Hospital, Beijing, China

**Keywords:** *KCNA2*, mosaic, epilepsy, encephalopathy, next generation sequencing

## Abstract

*KCNA2* gene mutations were described to cause a new molecular entity within the developmental and epileptic or epileptic encephalopathies. Here, we firstly reported a patient with an unusual mosaicism for *KCNA2*, presenting two distinct mosaic missense mutations at the same loci. Clinical trio-based whole-exome sequencing using next-generation sequencing (NGS) revealed two novel mutations in *KCNA2*: c.1225A > T [p.(Ile409Phe)] and c.1225A > C [p.(Ile409Leu)]. Both missense mutations were in mosaic status and Sanger sequencing confirmed them as *de novo*. The affected 5-year-old girl presented as seizures with fever sensitivity, and mild cognitive and behavioral disorders. EEG showed focal centrotemporal epileptiform discharges accompanied by nocturnal focal seizures at the age of slightly older than 5 years, more likely carrying a loss-of-function mutation of *KCNA2*-related phenotype. Further NGS with a mean coverage of 6950 × showed 26% (mosaic mutation reads/total reads) of the c.1225A > T mutation and 23% of the c.1225A > C mutation. The sum of their allele fractions was close to 50%, approximately equal to a heterozygous variant. The patient had no seizures for 8 months on combination of levetiracetam (18.75 mg/kg/d) and valproate (20 mg/kg/d) till the last follow-up at the age of 5 years and 11 months. Our findings highlighted the two mosaic mutations responsible for the pathogenesis of *KCNA2*-related encephalopathy. The patient expanded the mutational spectrum of *KCNA2*-related encephalopathy and provided new insight into the complex genetic disorder.

## Introduction

In 2015, mutation in *KCNA2* (MIM#176262) was firstly identified as a novel cause of developmental and epileptic or epileptic encephalopathy (Syrbe et al., 2015). The *KCNA2* gene encoded the voltage-gate K^+^ channel K_V_1.2 that belonged to the K_V_1 family with eight members expressed in the central nervous system (Long et al., 2005). Functional studies identified that pathogenic *KCNA2* mainly caused a dominant-negative loss-of-function and a drastic gain-of-function. Combined with the severity of the encephalopathy and the seizure disorder, the phenotype associated with *KCNA2* mutation could be differentiated into two main groups, the milder phenotype correlating with loss-of-function mutations and more severe phenotype with gain-of-function mutations (Syrbe et al., 2015). Subsequently, the phenotypic spectrum has been expanded up to include some forms of progressive myoclonus epilepsy or progressive ataxia, and myoclonic-atonic epilepsy (Pena and Coimbra, 2014; Canafoglia et al., 2019; Tang et al., 2020).

Mosaicism was defined as the presence of different genotypic mutations among cells of an individual derived from a single zygote (Forsberg et al., 2017). Depending on the timing of mutation acquisition, mosaicism might be restricted to the germline mosaicism (also known as gonadal mosaicism), somatic mosaicism and gonosomal mosaicism (a combination of germline and somatic mosaicism) (Biesecker and Spinner, 2013). The mosaicism arose due to postzygotic errors in DNA replication and could lead to disease state in the mosaic carriers or in the heterozygous offspring inheriting the mutant allele (Forsberg et al., 2017). The development and adoption of next-generation sequencing (NGS) opened up exciting possibilities for quantification of sequence mutations such that a high read depth could be enable to detect the low-level mosaic mutations. Thus, mosaicism had been increasingly reported in genes associated with epilepsy and neurodevelopmental disorders (Bartnik et al., 2011; Thiels et al., 2016; Liu et al., 2019; Zhang et al., 2019).

Here, we reported a case with two mosaic variants at the same nucleotide of the *KCNA2* gene, leading to two different pathogenic missense changes associated with epileptic encephalopathy.

## Materials and Methods

To unravel the molecular cause of the disease, trio-based exome sequencing was performed at the local genetic institutes using NGS techniques according to standards procedures. Genomic DNA was extracted from peripheral blood. NGS was performed on exon targets captured to an average target depth of 159× with over 95% of targeted regions, reaching a minimum coverage of 20×. Inheritance pattern, *in silico* predictions, control database [including the Exome Aggregation Consortium (ExAC), Exon Mutation Sever, 1000 Genomes database, and dbSNP], clinical laboratory reports, and findings in the literature were used to assess pathogenicity. Functional impact of mutations was predicted by three different *in silico* tools, Polyphen2^1^, SIFT^2^, MutationTaster^3^, and the Varsome database^4^. Mutations considered pathogenic or likely pathogenic were conformed via Sanger sequencing using standard methods.

For mutations in autosomal genes and X-linked genes, a mutation was considered possibly mosaic if it is observed at a lower-than-expected ratio of mutation to wild-type NGS reads and exhibited unequal amplification by Sanger sequencing. For further validated mosaic mutations in the affected patient, targeted NGS achieving a mean coverage of approximately 6950× with 100% targeted regions was performed.

We reviewed history obtained from medical records and from families via phone, including gender, age at seizure onset, seizure types, perinatal and personal history, family history, treatment and other relevant clinical data. Electroencephalogram (EEG), brain magnetic resonance and neuropsychological assessment, were reviewed.

Written informed consent was obtained from the legal guardians (parents) of the patient for diagnostic procedures and NGS. The study has been approved by the Ethical Committee of Peking University First Hospital.

## Case Presentation

The patient was a 5-year-old girl. She was born at 37 weeks of gestation. During the pregnancy, her mother had a history of two risk of miscarriage treated with progesterone. She had not previously been diagnosed with infertility and had not undergone any assisted reproductive technology procedures. The patient was delivered by cesarean section with a birth weight of 2000 g. She did not require resuscitation. She was the first child of healthy non-consanguineous parents. There was no familial history of any neuropsychiatric disease including epilepsy or febrile seizures.

At the age of 18 months, she presented with a febrile generalized tonic-clonic seizure (GTCS) for about 2 min associated with herpangina. After that, she had recurrent febrile GTCS for seven times with the duration of 1–2 min and also had a fever without an attack in rare cases. At the age of 5 years and 3 months, she developed the first afebrile seizure, presenting with focal motor seizure with secondarily GTCS for about 2 min at sleep onset. She had a mildly delayed developmental milestone, especially in gross and fine motor skills. Great progress was made after effective training. Developmental regression was noticed after seizures. Now, at the age of 5 years and 11 months, there was no significant delay in gross motor and language skills. But the significant cognitive impairments were noticed, including memory problems, learning disorders, attention span and irritability. She could not complete the simple algorithm even less than ten.

**FIGURE 1:**
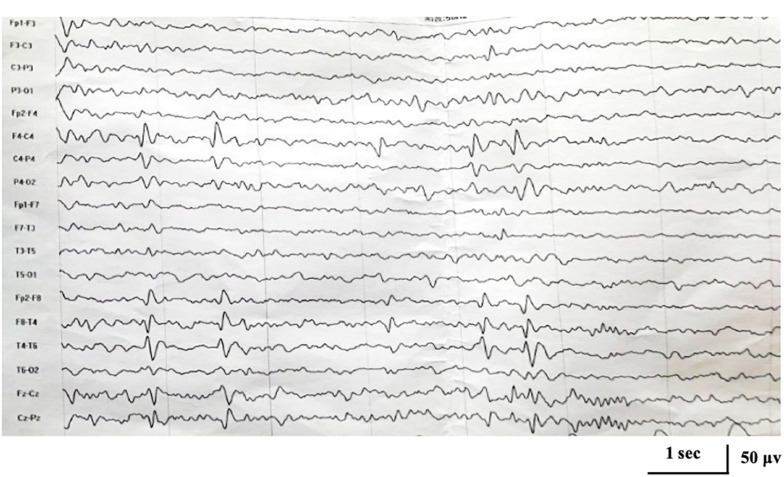
Interictal electroencephalogram demonstrating focal epileptiform discharges in centrotemporal area at the age of 5 years and 1 month.

No abnormality was found on the EEG at onset. At the age of 5 years and 1 month, EEG showed focal epileptiform discharges in centrotemporal area with activation during sleep (Figure 1), and brain magnetic resonance imaging was normal.

Rescue treatment with benzodiazepines (2.5 mg) was occasionally used by her parents when the patient had a fever and no seizure occurred. Levetiracetam treatment (33 mg/kg/d) was used at the age of 5 years and 2 months because of frequent febrile seizures and EEG abnormality. After a month, the dosage of levetiracetam was gradually increased to 50 mg/kg/d due to the first afebrile seizure. Later, because of side effects (hyperexcitability and poor sleep), the dosage of levetiracetam was reduced and valproate was added. The patient had no seizures for 8 months on combination of levetiracetam (18.75 mg/kg/d) and valproate (20 mg/kg/d) till the last follow-up at the age of 5 years and 11 months.

**FIGURE 2:**
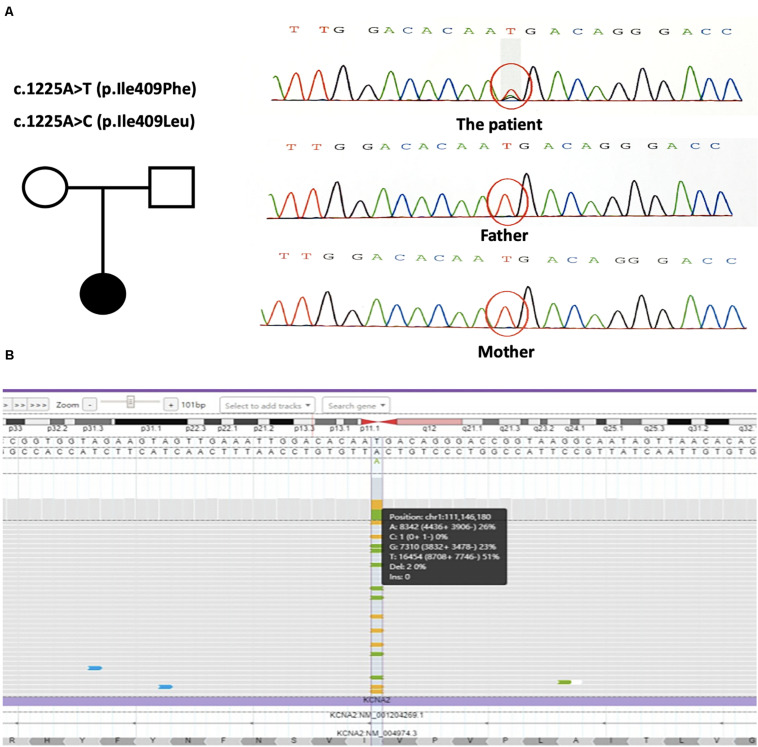
**(A)** The genetic map and the mutations sequence chromatograms in the *KCNA2* gene. Black circle: affected mutation-carrying female; white circle: female without *KCNA2* mutation; white square: male without *KCNA2* mutation. The red circles indicated the location of the identified mutations. **(B)** Visualization of the output bam file on Integrate Genome Viewer software tool. *KCNA2* gene region around the mutations c.1225A > T and c.1225A > C was analyzed with a 6950 × coverage; the sequence was shown in the forward strand. Vertical dotted lines showed the position relative to the missense mutations.

Trio-based whole-exome sequencing revealed two novel changes in exon 3 of the *KCNA2* gene: c.1225A > T [p.(Ile409Phe)] and c.1225A > C [p.(Ile409Leu)] [NM_004974]. Both missense mutations were in a mosaic status. NGS with a mean coverage of 159 × showed 31% (mosaic mutation reads/total reads) of the c.1225A > T mutation and 19% of the c.1225A > C mutation. Sanger sequencing confirmed the presence of the mutations in the patient, but not in her parents, characterizing them as *de novo* (Figure 2A). Both variants were classified as likely pathogenic using ACMG criteria (see Varsome) (Richards et al., 2015; Kopanos et al., 2019). Further NGS data at the loci of the mutations with a mean coverage of 6950 × showed 26% of the c.1225A > T mutation and 23% of the c.1225A > C mutation (Figure 2B).

## Discussion

We firstly reported here mosaicism of a 5-year-old girl presenting with two novel missense mutations in *KCNA2*. This patient was unusual in that there were two different mosaic mutation alleles at the same nucleotide of c.1225. NGS with a mean coverage of 159 × revealed 31% of the c.1225A > T mutation and 19% of the c.1225A > C mutation. And NGS with higher read depth at a mean coverage of 6950 × showed 26% of the c.1225A > T mutation and 23% the c.1225A > C mutation.

As a recently discovered gene associated with epilepsy disorders, more than sixty cases bearing over ten different *KCNA2* mutations had been reported (Masnada et al., 2017; Gong et al., 2020). The great majority of the mutations were missense and located in exon 3. Two main phenotypes emerged from the described cases (Syrbe et al., 2015; Masnada et al., 2017). A milder phenotype associated with loss-of-function mutations comprised infantile/early childhood seizure onset, frequent febrile and afebrile focal seizures. The c.1214C > T mutation was reported to be the most common loss-of-function mutation. A more severe phenotype carrying gain-of-function mutations presented as epilepsy, ataxia and intellectual disability. The c.1120A > G mutation was the most common gain-of-function mutation (Syrbe et al., 2015). Masnada et al. (2017) found that some of the gain-of-function mutations also showed some additional loss-of-function effects and proposed to subgroup those patients carrying mutations with similar electrophysiological properties (Masnada et al., 2017). The three phenotypic groups share some common clinical features.

Here, we report a case of *KCNA2*-related encephalopathy with two novel mutations located in a highly conserved and functionally important protein region. The mutations disrupted the pore domain of the voltage-gated potassium channel Kv1.2, which was thought to link the gate to the voltage-sensor (Holmgren et al., 1998; Long et al., 2005). Both mutations were neither found in ExAC nor 1000G databases. Mosaic mutations c.1225A > T/C [p.(Ile409Phe/Leu)] were both predicted as damaging by different prediction programs. Functional study of c.1214C > T [p.(Pro405Leu)] found a dramatic reduction of current amplitudes and thus a clear a loss of channel function (Syrbe et al., 2015). Due to similar clinical and EEG characteristics, it speculated that the mutations of c.1225A > T/C might also cause a loss-of-function effects. The sum of the allele fractions of the two mutant alleles was closed to 50%, approximately equal to heterozygous missense variants. This was consistent with the fact that *KCNA2*-related encephalopathy is an autosomal dominant disease (Syrbe et al., 2015). Genotype-phenotype analysis was performed. Clinically, our patient showed an age at onset in early childhood, fever sensitivity of seizures, and mild cognitive and behavioral disturbances, which were consistent with what had been previously reported in association with *KCNA2* heterozygous mutation. Taken together, mosaic mutations c.1225A > T/C were considered to be the pathogenic cause of the disease in our patient. Furthermore, interictal EEG in patients with loss-of-function mutations, especially c.1214C > T, had been reported to showed a peculiar pattern characterized by focal, mainly central or posterior-temporal epileptiform discharges with dramatic activation during sleep (Masnada et al., 2017; Gong et al., 2020). And among the 12 reported patients with c.1214C > T mutation, ten were presented with electrical status during sleep (ESES), diagnosed as atypical benign childhood epilepsy with centrotemporal spikes (BECT). In line with previous study, EEG in our case showed focal centrotemporal epileptiform discharges at the age of 5 years and 1 month, accompanied by nocturnal focal seizures. Therefore, our patient was more likely carrying a mutation with loss-of-function. Whether the patient would develop into atypical BECT with ESES required long-term follow-up.

Increasing evidence suggested that mosaic mutations had counted for a certain fraction of monogenic disorders, well known in tumorigenesis and overgrowth syndromes (Erickson, 2014; Tate et al., 2019). Recently, case reports had shown mosaicism in certain epilepsy-related and neurodevelopmental genes (Nakayama et al., 2018; Liu et al., 2019; Zhang et al., 2019). Stosser et al. (2018) firstly performed a systematic study of the extent and level of mosaicism detectable by NGS for patients with epilepsy (Stosser et al., 2018). They indicated that mosaic pathogenic mutations were identified frequently in nine genes associated with various neurological conditions, including *CDKL5*, *GABRA1*, *GABRG2*, *GRIN2A*, *GRIN2B*, *KCNQ2*, *MECP2*, *PCDH19*, *SCN1A*, and *SCN2A*. Due to the small number of cases diagnosed with *KCNA2* gene, this was the first description of mosaicism in only one patient.

As far as we knew, only two other patients with two mutant alleles had been reported to date (Eyries et al., 2012; Boutry-Kryza et al., 2014). Boutry-Kryza et al. (2014) described a 4-year-old girl presenting two distinct exonic deletions of the *CDKL5* gene in a mosaic state. And they proposed to explain the co-occurrence of these two events by the Fork Stalling and Template Switching mechanism (Boutry-Kryza et al., 2014). In the other case with hereditary hemorrhagic telangiectasia, it involved the presence of two adjacent heterozygous deleterious mutations. Both the two mutations were located within the same hairpin structure and the author speculated a template switching model might be responsible for the abnormalities (Eyries et al., 2012). In our case, we speculated that both mutations arose on the same chromosome, although the mechanism behind their formation remains unclear. The complex mosaicism might lead to underdiagnosis by routine techniques and brought a challenge to classifying pathogenicity of missense variants. In the future, the complex mosaicism in rare and genetic heterogeneous neurodevelopmental disorders should be considered in the study for new methods to interpret variant data.

The use of NGS is nowadays allowing the detection of unexpected genetic mosaicisms in genetic disease. Although we could analyze blood only, we are confident the same mosaicism was present in our patient brain, since others demonstrated that often mosaic mutations have allele frequencies similar in multiple tissues (Yang et al., 2017; Huang et al., 2018).

## Conclusion

Here was the first known instance of mosaicism in the *KCNA2* gene. Our patient expanded the mutational spectrum of *KCNA2*-related encephalopathy. Moreover, the findings of this study opened a new perspective for the unusual mosaicism of two distinct mutations in *KCNA2*-related encephalopathy and provided new insight into the complex genetic disorder. It highlighted the complexity of genomic events occurring during early embryogenesis and the consequences of mutational mosaicism upon pathogenic variability.

## Data Availability Statement

The raw data supporting the conclusions of this article will be made available by the authors, without undue reservation.

## Ethics Statement

The studies involving human participants were reviewed and approved by the Ethical Committee of Peking University First Hospital. Written informed consent to participate in this study was provided by the legal guardians (parents) of the patient. Written informed consent was obtained from the legal guardians (parents) of the patient for the publication of any potentially identifiable images or data included in this article.

## Author Contributions

ZY conceptualized and designed the study, coordinated the study overall, and revised the manuscript. PG co-designed the study, drafted the initial manuscript, and revised the manuscript. XJ and YZ helped to collect and summarize data and revised the manuscript. ZY, PG, XJ, and YZ approved the final revision of the article. All authors contributed to the article and approved the submitted version.

## Conflict of Interest

The authors declare that the research was conducted in the absence of any commercial or financial relationships that could be construed as a potential conflict of interest.

## Footnotes

1 http://genetics.bwh.harvard.edu./pph2/

2 https://sift.bii.a-star.edu.sg/

3 http://www.mutationtaster.org/

4 https://varsome.come/
